# Early relaparotomy in recipients after living donor liver transplantation: causes, risk factors, and consequences

**DOI:** 10.55730/1300-0144.5863

**Published:** 2024-06-06

**Authors:** Bora BARUT, Cengiz CEYLAN, Akile ZENGİN, Mehmet GÜZEL, Yasin DALDA, Sezai YILMAZ

**Affiliations:** 1 Department of General Surgery, Faculty of Medicine, İnönü University, Malatya, Turkiye; 2 Department of Gastroenterology Surgery, Eskişehir State Hospital, Eskişehir, Turkiye; 3 Department of Gastroenterology Surgery, Faculty of Medicine, Eskişehir Osmangazi University, Eskişehir, Turkiye; 4 Department of Gastroenterology Surgery, Malatya Training and Research Hospital, Malatya, Turkiye

**Keywords:** Early, relaparotomy, recipients, living donor, liver transplantation

## Abstract

**Background/aim:**

Despite advancements in surgical methodologies and the extensive perioperative and postoperative care administered to recipients, the prevalence of complications requiring early relaparotomy following living donor liver transplantation (LDLT) remains persistent. This study sought to analyze the determinants influencing relaparotomy occurrences in the initial 30 days following LDLT. Additionally, it was aimed to evaluate the impact of early laparotomy on both graft and patient survival within this distinct patient cohort.

**Materials and methods:**

The study encompassed recipients (n = 535) aged 18 years and older who underwent primary LDLT at our institution from January 2019 to December 2021. Exclusion criteria involved patients necessitating early retransplantation. Early relaparotomy was specified as surgical intervention within the initial 30 days following LDLT.

**Results:**

The study enrolled a total of 535 patients, among whom 85 (15.9%) underwent early relaparotomy. The median age of the patients was 54 (range: 41–60) years, with a predominant male representation (66.2%). Univariate analysis comparing the laparotomy and nonrelaparotomy groups revealed statistically significant differences in the creatinine (p = 0.043) and sodium (p = 0.025) levels, graft side (p < 0.001), etiology (p = 0.005), and blood loss (p = 0.012).

In the multivariate analysis, creatinine (p = 0.039; OR = 1.668; 95% CI = 1.027–2.709) and left lobe graft (p < 0.0001; OR = 3.611; 95% CI = 1.960–6.652) emerged as independent risk factors for relaparotomy.

**Conclusion:**

The primary causes of early relaparotomy following LDLT include postoperative bleeding, biliary leakage, and vascular complications. Preoperative elevation in creatinine and sodium levels, the presence of Budd–Chiari syndrome, utilization of a left lobe graft, and intraoperative blood loss are identified as risk factors associated with early relaparotomy after LDLT. Patients undergoing early relaparotomy exhibit inferior survival rates compared to those who do not.

## 1. Introduction

Living donor liver transplantation (LDLT) is widely acknowledged as an alternative to deceased donor liver transplantation (DDLT), particularly in regions with a relatively low number of available DDLTs. Despite advancements in surgical techniques and intraoperative/postoperative management, recipients undergoing LDLT continue to face persistent challenges with early relaparotomy, resulting in unfavorable survival outcomes [1]. The inherent technical complexities arise from the partial nature of LDLT grafts, characterized by relatively small diameters of vessels and bile ducts. Recent reports indicate higher relaparotomy rates in LDLT compared to DDLT. Early relaparotomy following LDLT is further associated with increased morbidity, mortality, and compromised graft and patient survival [2–4]. This study aimed to elucidate the causes, risk factors, and outcomes associated with early relaparotomy after LDLT.

## 2. Materials and methods

### 2.1. Study design and patients

This retrospective case-control study was carried out at a tertiary care center, encompassing data from all individuals who underwent liver transplantation procedures at our facility from January 2019 to December 2021. Liver transplants, postoperative follow-ups, management of postoperative complications, and relaparotomies are performed by senior liver transplant surgeons. The primary objective of this study was to discern factors contributing to relaparotomy within the initial 30 days following LDLT and ascertain the impact of early laparotomy on both graft and patient survival in this specific patient population. The study included individuals aged 18 years or older who underwent LDLT.

To ascertain risk factors for early laparotomy following LDLT in recipients, various clinical parameters were analyzed, including the recipient’s age, sex, body mass index (BMI), Model for End-Stage Liver Disease-Na score (MELD-Na), and preoperative creatinine (mg/dL), total bilirubin (mg/dL), albumin (g/dL), platelet (10^3^/μL), and international normalized ratio (INR) levels. Other factors such as a surgical history, the operation duration, intraoperative bleeding volume, ascites volume, length of hospital stay, etiology of liver disease, donor age and sex, biliary anastomosis type, graft type, graft-to-recipient weight ratio (GRWR), warm ischemia time (minutes), cold ischemia time (minutes), number of graft bile ducts, and number of graft hepatic arteries and portal vein were also examined. Furthermore, the study assessed the impact of early laparotomy on both graft and patient survival. Data were extracted from the institutional database, and ethical approval was obtained from the institutional review board with a waiver of informed consent (Date: 2023, Number: 5409).

### 2.2. Patient and donor selection

Dynamic computerized tomography scans were utilized for the assessment of intrahepatic malignancies and the evaluation of vascular structures in the recipients. Asymptomatic patients with MELD-Na scores exceeding 15 and decompensated patients (experiencing uncontrolled ascites, variceal bleeding, etc.) with MELD-Na scores below 15 underwent liver transplantation. Preoperatively, the appropriate graft weight was calculated based on the recipients’ body weights. Compatibility of the healthy donors’ blood types (A, B, and O) was verified. Hepatosteatosis rates were determined through magnetic resonance imaging, with patients exhibiting rates below 15% considered suitable. Intraoperatively, frozen sections were obtained from both lobes of the donor’s liver to confirm the hepatosteatosis rates. Preferably, the remaining liver in the donor was maintained at more than 30% of the total liver weight.

### 2.3. Statistical analysis

The sample size for this study was determined via power analysis utilizing G-Power 3.1 software. A power (1–β) of 0.90 and a 95% confidence interval (CI) were taken into account, yielding a calculated sample size of 76 participants per group. Consequently, a minimum total sample size of 152 participants was established for both groups. The normality of the distribution was assessed employing the Kolmogorov–Smirnov test. Nonparametric tests, specifically the Mann–Whitney U test, were applied as appropriate. Descriptive statistics, including median values and the interquartile range (IQR), were reported for the variables. The optimal cut-off values for creatinine, sodium, and blood loss were determined using receiver operating characteristic (ROC) curve analysis. Categorical variables underwent analysis using chi-squared analysis, and the results were presented as frequencies and percentages. Subsequently, a prospective selective multivariate logistic regression analysis was conducted to examine variables demonstrating statistical significance. The adequacy of fit for the logistic regression model was evaluated using the Hosmer–Lemeshow test. Statistical significance was accepted as p < 0.05.

## 3. Results

The study enrolled a total of 535 patients, with 85 recipients (15.9%) undergoing early relaparotomy, performed 91 times (two times in four patients; two patients underwent surgery due to intraabdominal hemorrhage, one patient due to hepatic artery thrombosis, and one patient due to biliary peritonitis and three times in two patients; two patients underwent surgery due to luminal organ perforation. The mean relaparotomy time was 6.8 days (range: 0–30). The median age of the patients was 54 (range: 41–60) years, and they were predominantly male (66.2%). Early relaparotomy was necessitated by various factors, including postoperative bleeding (n = 30, 35.2%), vascular pathology [(n = 22, 25.8%) comprising hepatic artery thrombosis (n = 10), portal vein thrombosis (n = 8), hepatic venous outflow failure (n = 3), and hepatic artery thrombosis + portal vein thrombosis (n = 1)], biliary peritonitis (n = 13, 15.2%), and other causes (n = 20, 23.5%). The recipients had a median BMI of 26 kg/m^2^ (range: 23–29). Regarding the donors, the median age was 29 (range: 24–36) years, with 188 (35.1%) females and 347 (64.9%) males. Demographic data for the patients and donors are summarized in Table 1.

In the univariate analysis comparing the laparotomy and nonrelaparotomy groups, statistically significant differences were noted in the creatinine (p = 0.043) and sodium (p = 0.025) levels, graft side (p < 0.001), etiology (p = 0.005), and blood loss (p = 0.012). Specifically, patients with Budd–Chiari syndrome and those who received a left graft demonstrated a higher likelihood of undergoing early relaparotomy, with the percentage to be specified. The cut-off values for creatinine (<0.72 mg/dL), sodium (>136 mmol/L), and amount of bleeding (>550 mL) were determined through ROC analysis, as outlined in Table 2. In the multivariate analysis, creatinine (p = 0.039; OR = 1.668; 95% CI = 1.027–2.709) and left lobe graft (p < 0.0001; OR: 3.611; 95% CI: 1.960–6.652) emerged as independent risk factors for relaparotomy (Table 3).

Among the patients undergoing relaparotomy, the 1, 3, and 12-month survival rates were 76.5%, 72.9%, and 65.9%, respectively. In the nonrelaparotomy group, the corresponding rates were 96%, 93.3%, and 90.7%, respectively. Early relaparotomy was correlated with diminished patient survival, and a statistically significant difference between the two groups was observed (p < 0.001), as detailed in Table 4.

## 4. Discussion

Early relaparotomy following LDLT represents a critical condition that can lead to premature graft failure and patient mortality. Literature reports indicate relaparotomy rates after LDLT ranging from 9.2% to 34% [5]. In the present study, the early relaparotomy rate after LDLT was 15.9%, aligning with the reported range in the existing literature.

Postoperative bleeding, vascular complications, biliary complications, and intraabdominal infections stand as the most prevalent causes for early relaparotomy following LDLT [6,7]. In the present series, the three most frequent reasons for early relaparotomy were bleeding, vascular complications, and biliary peritonitis, mirroring findings in the existing literature. Previous studies have indicated that the rate of early relaparotomy after LDLT attributed to bleeding ranges from 40% to 50% [2,3]. This complication often stems from preoperative prolonged coagulopathy and surgical techniques [8]. In the current study, early relaparotomy due to postoperative bleeding was performed in 30 (35.2%) patients, and no statistically significant difference was observed between the early relaparotomy and nonearly relaparotomy groups concerning the preoperative platelet counts and INR levels.

The literature reports a range of 20% to 40% for the rate of early relaparotomy related to biliary complications [4,9]. In the current study, early relaparotomy attributable to biliary leakage occurred in 15.2% of the patients. This comparatively lower rate, in contrast to the literature, was attributed to the utilization of interventional radiology practices at our institute.

Another cause for early relaparotomy after LDLT is vascular complications, including hepatic artery thrombosis, portal vein thrombosis, and hepatic venous outflow failure, with reported rates ranging from 19.2% to 27.3% in the literature [10]. In the current study, vascular complications were observed in 22 (25.8%) patients. Specifically, hepatic artery thrombosis was identified in 10 (45.4%) patients, portal vein thrombosis in eight (36.3%) patients, and hepatic venous outflow failure in three (13.6%) patients. Additionally, one patient presented with both hepatic artery and portal vein thrombosis.

In the literature, various risk factors have been identified for early relaparotomy after LDLT. Previous studies have indicated that a history of upper abdominal surgery, high MELD-Na score, presence of massive ascites, coagulopathy, intraoperative blood loss requiring transfusion, prolonged operation time, severe illness, hepatic encephalopathy, and insufficient portal venous flow are associated with an increased risk of early relaparotomy [2–5,11]. In the present study, both univariate and multivariate analyses revealed that preoperative creatinine and sodium levels, Budd–Chiari syndrome, use of the left lobe graft, and intraoperative blood loss are independent risk factors for early relaparotomy after LDLT.

Early relaparotomy following LDLT is linked to an extended hospital stay, compromised graft survival, and diminished patient survival. Patients undergoing early relaparotomy after LDLT experience a three-fold increase in mortality rates compared to those without the need for reoperation [12,13]. In the current study, the early relaparotomy group exhibited inferior 1, 3, and 12-month patient survival rates in comparison to the nonrelaparotomy group.

In summary, early relaparotomy after LDLT is predominantly necessitated by postoperative bleeding, biliary leakage, and vascular complications. Identified risk factors for early relaparotomy encompass preoperative derangements in creatinine and sodium levels, the presence of Budd–Chiari syndrome, utilization of the left lobe graft, and intraoperative blood loss. Furthermore, patients undergoing early relaparotomy exhibit diminished survival rates compared to their counterparts who do not undergo such interventions.

## Figures and Tables

**Table 1:** Demographic data of recipients and donors.

Variables		Median (IQR)	Count (%)
Age, years		54 (41–60)	
Sex	Female		181 (33.8%)
	Male		354 (66.2%)
Recipient BMI, kg/m^2^		26 (23–29)	
Donor age, years		29 (24–36)	
Donor sex	Female		188 (35.1%)
	Male		347 (64.9)
MELD-Na^a^ score		14 (10–20)	
Preoperative etiology	Malignancy		61 (11.4%)
	Viral		157 (29.3%)
	Wilson+Metabolic diseases		16 (3.0%)
	PBC^b^, PSC^c^, Autoimmune hepatitis		53 (9.9%)
	Cryptogenic		161 (30.1%)
	Ethanol		24 (4.5%)
	Fulminant		14 (2.6%)
	Budd–Chiari		29 (5.4%)
	NASH^d^		7(1.3%)
	Others		13(2.4%)
Reoperation	Presence		85 (15.9%)
	Absence		450 (84.1%)

a MELD-Na: Model for End-Stage Liver Disease-Na,

b primary biliary cirrhosis,

c primary sclerosing cholangitisi,

d NASH: nonalcoholic steatohepatitis.

**Table 2:** Univariate analysis between relaparotomy and nonrelaparotomy groups.

		Relaparotomy		Nonrelaparotomy		
Variables		Median (IQR)	Count (%)	Median (IQR)	Count (%)	p
Age, years		52 (37–60)		54 (60–43)		0.172
Sex	Female		32 (37.6%)		149 (33.1%)	0.418
	Male		53 (62.4%)		301 (66.9%)	
Recipient BMI^a^, kg/m^2^		26 (23–29)		26 (29–23)		0.694
Donor age, years		31 (37–25)		29 (36–23)		0.165
Donor sex	Female		34 (40.0%)		154 (34.2%)	0.36
	Male		51 (60.0%)		296 (65.8%)	
MELD-Na^b^ score		13 (20–10)		14 (20–10)		0.779
Creatinine, mg/dL		70 (0.90–0.60)		0.80 (0.94–0.70)		0.043
Albumin, g/dL		2.90 (3.50–2.40)		2.80 (3.30–2.40)		0.429
Preop.^c^ total bilirubin, mg/dL		2.54 (5.30–1.50)		2.40 (5.00–1.34)		0.650
Preop. sodium, mmol/L		137 (139–134)		136 (138–132)		0.025
Preop. platelet, 10^3^/uL		113 (208–70)		105 (163–70)		0.203
Preop. INR^d^		1.39 (1.66–1.20)		1.39 (1.62–1.20)		0.648
Operation (history)	Presence		7 (8.2%)		24 (5.3%)	0.294
	Absence		78 (91.8%)		426 (94.7%)	
Biliary anastomosis type	Duct to duct		82 (96.5%)		439 (98.0%)	0.386
	Hepaticojejunostomy		3 (3.5%)		9 (2.0%)	
Graft side	Right		63 (74.1%)		412 (91.8%)	<0.001
	Left		22 (25.9%)		37 (8.2%)	
GRWR^e^		10 (12–9)		10 (12–9)		0.649
Warm ischemia time (min)		53 (69–38)		53 (66–41)		0.832
Cold ischemia time (min)		99 (131–78)		95 (118–75)		0.156
Operation duration (min)		540 (600–480)		518 (580–480)		0.341
Blood lose (cc)		500 (1000–400)		500 (800–350)		0.012
Ascites amount (cc)		1200 (4400–0)		500 (3500–0)		0.102
Etiology	Budd–Chiari		10 (11.8%)		19 (4.2%)	0.005
	Other		75 (88.2%)		431 (95.8%)	
Graft duct count	Single		61 (71.8%)		307 (68.2%)	0.518
	Multiple		24 (28.2%)		143 (31.8%)	
Graft arteria count	Single		80 (94.1%)		440 (97.8%)	0.061
	Multiple		5 (5.9%)		10 (2.2%)	
Graft porta count	Single		84 (98.8%)		448 (99.6%)	0.406^*^
	Multiple		1 (1.2%)		2 (0.4%)	

a BMI: body mass index,

b MELD-Na: Model for End-Stage Liver Disease-Na,

c Preop.: preoperative,

d INR: international normalized ratio,

e GRWR: graft-to-recipient weight ratio.

**Table 3:** Multivariate analysis for identified as independent risk factors for relaparotomy.

Variables	OR	95% CI	*p-*value
Etiology (Budd–Chiari)	2.148	0.904–5.103	0.083
Graft side LEFT side	3.611	1.960–6.652	<0.001
Blood amount > 550 mL	1.511	0.927–2.461	0.098
Creatinine < 0.72 mg/dL	1.668	1.027–2.709	0.039
Sodium > 136mmol/L	1.375	0.841–2.247	0.204

OR: odds ratio.

**Table 4:** The survival rates of patients based on the need for relaparotomy.

	Relaparotomy	Nonrelaparotomy			
Survival	Count (%)	Count (%)	OR	95% CI	*p*-value
Patient survival 1 month	65 (76.5%)	432 (96%)	7.39	3.71–14.7	<0.001
Patient survival 3 month	62 (72.9%)	420 (93.3%)	5.19	2.84–9.51	<0.001
Patient survival 12 month	56 (65.9%)	408 (90.7%)	5.03	2.9–8.72	<0.001

OR: odds ratio, CI: confidence interval.
